# *Staphylococcus aureus* in the Community: Colonization Versus Infection

**DOI:** 10.1371/journal.pone.0006708

**Published:** 2009-08-20

**Authors:** Maureen Miller, Heather A. Cook, E. Yoko Furuya, Meera Bhat, Mei-Ho Lee, Peter Vavagiakis, Paul Visintainer, Glenny Vasquez, Elaine Larson, Franklin D. Lowy

**Affiliations:** 1 Department of Epidemiology and Biostatistics, School of Public Health, New York Medical College, Valhalla, New York, United States of America; 2 Division of Infectious Diseases, Department of Medicine, Columbia University, College of Physicians & Surgeons, New York, New York, United States of America; 3 Panna Technologies, Inc., Brooklyn, New York, United States of America; 4 School of Nursing, Columbia University, New York, New York, United States of America; 5 Department of Pathology, Columbia University, College of Physicians & Surgeons, New York, New York, United States of America; Yale University, United States of America

## Abstract

**Background:**

Antibiotic-resistant *Staphylococcus aureus* infections have increased dramatically in the community, yet *S. aureus* nasal colonization has remained stable. The objectives of this study were to determine if *S. aureus* colonization is a useful proxy measure to study disease transmission and infection in community settings, and to identify potential community reservoirs.

**Methodology/Principal Findings:**

Randomly selected households in Northern Manhattan, completed a structured social network questionnaire and provided nasal swabs that were typed by pulsed field gel electrophoresis to identify *S. aureus* colonizing strains. The main outcome measures were: 1) colonization with *S. aureus*; and 2) recent serious skin infection. Risk factor analyses were conducted at both the individual and the household levels; logistic regression models identified independent risks for household colonization and infection.

**Results:**

321 surveyed households contained 914 members. The *S. aureus* prevalence was 25% and MRSA was 0.4%. More than 40% of households were colonized. Recent antibiotic use was the only significant correlate for household colonization (p = .002). Seventy-eight (24%) households reported serious skin infection. In contrast with colonization, five of the six risk factors that increased the risk of skin infection in the household at the univariate level remained independently significant in multivariable analysis: international travel, sports participation, surgery, antibiotic use and towel sharing. *S. aureus* colonization was not significantly associated with serious skin infection in any analysis. Among multiperson households with more than one person colonized, 50% carried the same strain.

**Conclusions/Significance:**

The lack of association between *S. aureus* nasal colonization and serious skin infection underscores the need to explore alternative venues or body sites that may be crucial to transmission. Moreover, the magnitude of colonization and infection within the household suggests that households are an underappreciated and substantial community reservoir.

## Introduction

In the past decade there has been a dramatic increase in community-associated (CA) *Staphylococcus aureus* infections, many due to methicillin-resistant strains [1], [2].

With the exception of *S. aureus* outbreaks in high-risk settings, little is known about the reservoirs or settings through which individuals in the community are exposed to *S. aureus*. While numerous hospital studies have demonstrated the role of *S. aureus* nasal colonization as a risk factor for subsequent infection with the colonizing strain [3], [4], the role of nasal colonization in the community is less well established. To date community-based studies have yielded conflicting results on the role of *S. aureus* nasal colonization as a risk factor for subsequent infection [5], [6], [7], [8]. Moreover, community-based research has not consistently examined the relationship between potential *S. aureus* risk factors and serious skin infection, and instead often relies on *S. aureus* nasal colonization as a proxy for infection.

In the hospital setting, there are two main environmental risk factors relevant to the community setting that contribute to *S. aureus* transmission: incomplete or ineffective hygienic practices and a high prevalence of *S. aureus*. If these factors are considered in the community setting, then two separate lines of inquiry are established. The first question addresses issues of hygiene and the types of community settings where hygienic practices may contribute to *S. aureus* transmission, such as the household. The second question is related to *S. aureus* prevalence within a well-defined population or environment that could constitute identifiable reservoirs.

If interventions are to be developed to disrupt the growing epidemic of multidrug-resistant (MDR) *S. aureus*, including methicillin resistant *S. aureus* (MRSA), then a comprehensive understanding of the types of contact, as well as the contexts of contact, that lead to *S. aureus* transmission is required. Therefore, this study was designed to estimate the population prevalence of *S. aureus* in a geographically well-defined community; to document *S. aureus* nasal colonization distribution and strain similarity; and to identify any community, health-related and hygiene risk factors for *S. aureus* colonization and serious skin infection, separately. In contrast with earlier studies, separate analyses are carried out at the individual and the household levels.

## Materials and Methods

### Ethics

Eligible index respondents who provided preliminary verbal consent were enrolled in the study and a home visit was scheduled. Upon arrival, interviewers obtained written informed consent from the index respondent and from all participating household members. Parental consent was required for the participation of children; pediatric assent was obtained from those capable of providing it. The study was reviewed and approved by the Columbia University Institutional Review Board.

### Setting

This population-based study was conducted in the Northern Manhattan community that serves as the catchment area for Columbia University Medical Center (CUMC), as defined by 13 US census tracts. The area is home to the largest Dominican community in the country, approximately 220,000.

### Sample selection

Random-digit dialing (RDD) was used to select households to screen for eligibility. Five attempts were made to contact an adult at each telephone number. Of the 1154 households successfully contacted, 553 (48%) agreed to participate, a statistic comparable to other published studies using RDD [9]. The individual who answered the phone was recruited as the index respondent (*i.e.*, the person who provided survey information for all household members). Eligible index respondents had to be resident in the household, age ≥18 years and willing to complete the survey on behalf of the household. Complete survey data and at least one nasal swab were available for 444 eligible households; 43 (7.8%) had incomplete interviews and 66 (11.9%) index respondents did not provide *S. aureus* specimens. All data were collected between February 2004 and June 2006.

### Study procedures

A standardized network questionnaire was administered to the index respondent, who provided data for all individuals currently living in the household. Questions regarding risk factors for *S. aureus* included health-related exposures (*e.g.*, antibiotic use). Serious skin infections requiring treatment at home or by a medical professional were assessed for the past six months and included skin abscesses or boils, skin ulcers that drained pus, and skin infections similar to the ones shown in the photo created for the questionnaire. Community exposures to *S. aureus* were assessed, including settings where household members spent more than casual time (*e.g.*, daycare) and activities such as sports. Data were collected at both the individual level (*e.g.*, age of each household member) and the household level (*e.g.*, household towel sharing). With the exception of household size, individual level data were also transformed into dichotomous household level variables (*e.g.*, the presence of children <18 years old).

Anterior nares cultures were collected (Becton Dickinson Culturette Systems, Sparks, MD) from all household members willing to participate, including children ≥1 year. Interviewers made up to three follow-up home visits to collect specimens from household members not present at the interview. Households received a nominal sum in appreciation for their participation.

### Microbiology

The swabs collected from subjects' anterior nares were cultured onto Mannitol Salt Agar (Becton Dickinson). Positive colonies were those that fermented mannitol and turned the agar yellow. Positive colonies were next isolated onto 5% Sheep Blood Agar plates (Becton Dickinson) and a single colony selected for further analysis. *Staphylococcus aureus* species identification was confirmed by StaphAurex (Danford, UK) [10]. Strains were identified as methicillin resistant using a previously descried PCR assay [11].

All positive isolates were compared by pulsed field gel electrophoresis (PFGE) and analyzed using BioNumerics software v.4.00 (Applied Maths). Pairwise similarity scores were calculated by the Dice coefficient and overall similarity score was calculated using the Unweighted Pair Group Method with Arithmetic mean (UPGMA, also known as the average linkage method). Isolates identified as methicillin-resistant by PCR were further analyzed for their specific Staphylococcal Chromosomal Cassette (SCC)*mec* type using the multiplex PCR assay and validated using the *ccr* gene complex primers as previously described [12], [13].

### Statistical Analysis

The 444 eligible households contained 1346 members. Of these, 1012 (75%) provided nasal swabs. Those who refused to provide swabs differed significantly from compliers only by gender; men were significantly more likely to refuse than women (p<.001). Refusers were distributed across households; complete data were available for all household members for 240 (54%) of 444 eligible households, resulting in a loss of power to detect relationships. In order to retain a larger portion of the data and to reduce any bias associated with refusal to provide specimens, households in which two thirds (≥67%) of respondents provided nasal swabs were included. This decision was based on a median household size of n = 3. Since the presence of *S. aureus* colonization in the household was dichotomized, a conservative assumption was made that if two thirds of household members provided negative specimens, the remaining third were also negative. Of the 321 (72%) of 444 eligible households with at least two thirds of individuals with complete data, there were 914 members, 91 (10%) of whom were missing specimen data. Index respondents in the 321 household were mostly female (260 or 81%), 271 (84%) were Latino and had a median age of 48. To assess for potential biases, all analyses were repeated on the subsample of 240 households with complete data. The estimates were virtually identical; therefore, results from the first set of analyses are presented.

Statistical analyses were first conducted to identify individual level (n = 914) correlates of *S. aureus* colonization and of skin infection. In order to control for the lack of independence of participants who were recruited as members of households, generalized estimating equations (GEE) were used. Analyses were repeated at the household level (n = 321) to examine potential *S. aureus* colonizing and skin infection pressures, since *S. aureus* may enter the household through one individual but be transmitted to another. In this set of analyses, logistic regression models were used to control for household size. Analyses were limited to an examination of the presence or absence of *S. aureus* within the household, rather than to the number or percent of *S. aureus*-positive specimens or serious skin infections within the household. Final logistic regression models with backward elimination identified independent household correlates of household *S. aureus* colonization and household skin infection, separately. Any community, health-related or hygiene variables found to be associated with household *S. aureus* colonization or household skin infection at the univariate level (p<.20) were included in the models [14], [15]. Adjusted odds ratios (aORs), 95% confidence intervals (CIs) and p-values are reported for both GEE and logistic regression analyses. Two tailed p-values are significant at p<.05. SAS version 9.1.3 was used for all statistical analysis (SAS Institute; Cary, NC).

## Results

### Characteristics of individual household members

Table 1 compares US census statistics for the 13 catchment area tracts with statistics for the 914 individuals included in the present analysis. The majority of eligible index respondents (260 or 81%) of the 321 surveyed households were female (mean age 48), as were a majority of the 914 household members. Almost 90% of study participants were Latino. The mean age of the sample was 33 years (sd 23).

**Table 1:** Comparison of US census data for Northern Manhattan with study participant data.

Characteristic	Census Data 255,589		Study Data 914	
	N	(%)	N	(%)
**Ethnicity**				
Hispanic*	173,755	(68)	813	(89)
Non Hispanic	81,834	(32)	101	(11)
**Gender**				
Male	120,866	(47)	362	(40)
Female	134,723	(53)	552	(60)
**Age**				
<5	17878	(7)	89	(10)
5–17	49196	(19)	238	(26)
18–44	112,195	(44)	297	(32)
45–64	51,091	(20)	190	(21)
65+	21,750	(9)	100	(11)

* There are 271 (84%) Hispanic households in the study database and 50 (16%) Non-Hispanic households.

### *S. aureus* prevalence and risk correlates

Among the 823 (90%) of 914 eligible household members who provided nasal specimens, 203 (25%) were *S. aureus* positive. Table 2 provides information on the background characteristics of individual household members that may be associated with increased *S. aureus* colonization. In addition, relationships between potential risk correlates and *S. aureus* colonization (controlling for lack of participant independence) are presented. Only male gender was significantly associated with *S. aureus* nasal colonization.

**Table 2:** Individual level risk correlates and *S. aureus* nasal colonization among 914 individuals from 321 Northern Manhattan households, controlling for household membership (univariate GEE analysis).

Characteristics	Total population		S. aureus positive N = 203 (25%)	*S. aureus* negative N = 620 (75%)	Univariate adjusted odds ratio		p-value
*Sociodemographics*	N	(%)	%	%	aOR	(95% CI)	
**Male**	362	(39.6)	30	70	1.6	(1.2, 2.2)	.003
**Female**	552	(60.4)	22	78			
**Latino**	813	(89.0)	25	75	0.9	(0.5, 1.8)	.84
**Other**	101	(11.0)	24	76			
**Age** a					–	---	.11
**<5**	84	(9.2)	15	85			
**5–17**	238	(26.2)	33	67			
**18–24**	74	(8.2)	29	71			
**25–44**	223	(24.5)	24	76			
**45+**	290	(31.9)	20	80			
**Employed**	286	(31.3)	23	77	1.1	(0.7, 1.6)	.68
**Unemployed**	628	(68.7)	25	75			
***Community exposures to S. aureus***							
**Job exposure**	58	(6.4)	13	87	2.2	(1.0, 5.1)	.06
**No job exposure**	856	(93.6)	26	74			
**International travel (within 6 mos)**	171	(18.7)	21	79	1.3	(0.8, 2.1)	.27
**No travel**	743	(81.3)	25	75			
**Sports participation (within 6 mos)**	213	(23.3)	23	77	1.1	(0.7, 1.5)	.80
**No sports**	701	(76.7)	25	75			
**In daycare**	87	(9.5)	16	84	1.9	(1.0, 3.7)	.06
**Not in daycare**	827	(90.5)	26	74			
***Health-related exposures to S. aureus***							
**Hospitalized** b **(2 yrs)**	143	(15.7)	26	74	0.9	(0.6, 1.3)	.60
**Not hospitalized**	771	(84.3)	24	76			
**Surgery (6 mos)**	51	(5.6)	20	80	1.2	(0.6, 2.4)	.47
**No surgery**	863	(94.4)	25	75			
**Antibiotic use (6 mos)**	213	(23.3)	22	78	1.1	(0.8, 1.7)	.47
**No antibiotic use**	701	(76.7)	26	74			
**Skin infection (6 mos)**	97	(10.6)	30	70	0.8	(0.5, 1.2)	.21
**No skin infection**	817	(89.4)	24	76			
**Self reported health Excellent/Good**					–	---	.59
**Fair**	621	(67.9)	25	75			
**Poor**	239	(26.2)	25	75			
	54	(5.9)	20	80			

a Missing 5.

b The top five reasons for hospitalization were cardiovascular disease (17%), infections (14%), childbirth (13%), respiratory conditions (12%), and surgeries (12%).

### Serious recent skin infections and risk correlates

Among the 914 household members, 97 (11%) reported serious skin infections in the past six months. Significant associations were identified between skin infections and age, ethnicity and health related exposures (Table 3). Of note, *S. aureus* nasal colonization was not significantly associated with skin infections.

**Table 3:** Individual level risk correlates and serious skin infection among 914 individuals from 321 Northern Manhattan households, controlling for household membership (univariate GEE analysis).

Characteristics	Total population			Skin infection N = 97 (11%)	No skin infection N = 817 (89%)	Univariate adjusted odds ratio			p-value
*Sociodemographics*	N	(%)		%	%	aOR	(95% CI)		
**Male**	362	(39.6)		12	88	1.3	(0.8,2.0)		.22
**Female**	552	(60.3)		10	90	1.3	(0.8,2.0)		.22
**Latino**	813	(89.0)		9	91	0.4	(0.2,0.8)		.006
**Other**	101	(11.0)		20	80	0.4	(0.2,0.8)		.006
**Age** a									
**<5**	84	(9.2)		20	80	-	-		.04
**5–17**	238	(26.2)		11	89				
**18–24**	74	(8.2)		7	93				
**25–44**	223	(24.5)		9	91				
**45+**	290	(31.9)		10	90				
**Employed**	286	(31.3)		10	90	0.9	(0.6,1.5)		.80
**Unemployed**	628	(68.7)		11	89				
***Community exposures to skin infection***									
**Job exposure**	58	(6.4)		10	90	1.0	(0.4,2.3)		.96
**No job exposure**	856	(93.6)		11	89				
**International travel (within 6 mos)**	171	(18.7)		15	85	1.3	(0.8,2.1)		.27
**No travel**	743	(81.3)		10	90				
**Sports participation (within 6 mos)**	213	(23.3)		14	86	1.5	(0.9,2.4)		.10
**No sports**	701	(76.7)		10	90				
**In daycare**	87	(9.5)		16	84	1.6	(0.9,3.0)		.10
**Not in daycare**	827	(90.5)		10	90				
***Health-related exposures to skin infection***									
**Hospitalized** b **(2 yrs)**	143		(15.7)	15	85	1.6	(1.0,2.6)	.07	
**Not hospitalized**	771		(84.3)	10	90				
**Surgery (6 mos)**	51		(5.6)	24	76	2.7	(1.4,5.3)	.003	
**No surgery**	863		(94.4)	10	90				
**Antibiotic use (6 mos)**	213		(23.3)	18	82	2.2	(1.4,3.5)	.001	
**No antibiotic use**	701		(76.7)	8	39				
***S. aureus*** ** positive**	97		(10.6)	14	86	0.8	(0.5,1.2)	.21	
***S. aureus*** ** negative**	817		(89.4)	11	89				
**(N = 823)**									
**Self reported health**						-	-	.45	
**Excellent/Good**	621		(67.9)	10	90				
**Fair**	239		(26.2)	10	90				
**Poor**	54		(5.9)	17	83				

a Missing 5.

b The top five reasons for hospitalization were cardiovascular disease (17%), infections (14%), childbirth (13%), respiratory conditions (12%), and surgeries (12%).

### *S. aureus* colonization at the household level

Among the 321 households, 138 (43%) had at least one member colonized with *S. aureus*. The mean and median household size was n = 3 (sd 2; range 1 to 8); larger household size was significantly associated with *S. aureus* colonization within the household (3.7 vs. 2.2 household members, p<.001). Table 4 reports the relationship between community, health-related and hygiene exposures, and household level *S. aureus* colonization, controlling for household size.

**Table 4:** Household level risk correlates for the presence of *S. aureus* nasal colonization in 321 Northern Manhattan households, controlling for household size.

Exposure categories	Total population		*S. aureus* positive N = 138 (43%)	*S. aureus* negative N = 183 (57%)		Univariate adjusted odds ratio	Multivariate adjusted odds ratio
	N	(%)	%	%		aOR (95% CI)	aOR (95% CI)
***Community exposures of household to S. aureus***							
**Employed household**	188	(58.6)	52	48		1.1	-
**Unemployed**	133	(41.4)	31	69		(0.6, 1.9)	
**Job exposure**	51	(15.9)	55	45		0.9	-
**No job exposure**	270	(84.1)	41	59		(0.5, 1.9)	
**International travel (within 6 mos)**	102	(31.8)	42	58		0.7	-
**No travel**	219	(68.2)	43	57		(0.4, 1.2)	
**Sports participation (within 6 mos)**	119	(37.1)	51	49		0.9	-
**No sports**	202	(62.9)	38	62		(0.5, 1.6)	
**Child in daycare**	64	(19.9)	59	41		0.8	-
**No child in daycare**	257	(80.1)	39	61		(0.4, 1.6)	
**Child <5 years old in home**	64	(19.9)	59	41		0.8	-
**No children <5 years old**	257	(80.1)	39	61		(0.4, 1.6)	
**Child <18 years old in home**	159	(49.5)	63	37		2.0	-
**No children <18 years old**	162	(50.5)	23	77		(1.0, 4.1)	
***Health-related exposures of household to S. aureus***							
**Hospitalization (2 yrs)**	124	(38.6)	46	54		0.9	-
**No hospitalization**	197	(61.4)	41	59		(0.5, 1.5)	
**Surgery (within 6 mos)**	48	(15.0)	46	54		1.0	-
**No surgery**	273	(85.0)	42	58		(0.5, 1.9)	
**Antibiotic use (within 6 mos)**	165	(51.4)	38	62		0.5	0.4
**No antibiotic use**	156	(48.6)	48	52		(0.3, 0.8)^**^	(0.2,0.7)^**^
**Skin infection (within 6 mos)**	78	(24.3)	54	46		1.4	-
**No skin infections**	243	(75.7)	40	60		(0.8, 2.4)	
***Hygiene-related exposures of household to S. aureus***							
**Shared towels**	60	(18.7)	58	42	1.5		-
**No shared towels**	261	(81.3)	39	61	(0.8, 2.8)		
**Shared razors**	36	(11.2)	53	47	1.1		-
**No shared razors**	285	(88.8)	42	58	(0.5, 2.3)		

* p≤.05, ^**^p<.01, ^***^p<.001.

Few households reported the presence of known risk factors for *S. aureus* colonization: a history of *S. aureus* infection (<1%), a history of injection drug use (2%), or an HIV-infected household member (<1%). Only recent antibiotic use by a household member was significantly protective against *S. aureus* colonization within the household. Sources of antibiotics for the household were assessed: 232 (72%) had ever acquired antibiotics with a prescription, 55 (17%) from family, friends or other household leftovers, and 68 (21%) from another source, including local botanical shops or bodegas, and international sources.

In the final backward elimination logistic regression model, only the protective effect of household antibiotic use remained statistically significant in a model that initially included international travel, children <18 years old and towel sharing.

### Serious recent skin infection at the household level

Among the 321 households, 78 (24%) reported serious skin infection. Risk factors in all three exposure categories (i.e., community, health related and hygiene related) were significantly associated with increased risk of serious skin infection within the household (Table 5). In the final backward elimination logistic regression model, five of six correlates found to significantly increase the risk of serious skin infection at the univariate level remained independently associated.

**Table 5:** Household level risk correlates for the presence of serious skin infection in 321 Northern Manhattan households, controlling for household size.

Exposure categories	Total population			Skin infection N = 78 (24%)	No skin infection N = 243 (76%)	Univariate adjusted odds ratio	Multivariate adjusted odds ratio
	N	(%)		%	%	aOR (95% CI)	aOR (95% CI)
***Community exposures of household to skin infection***							
**Employed household**	188	(58.6)		30	70	1.8	-
**Unemployed**	133	(41.4)		16	84	(1.0,3.4)	
**Job exposure**	51	(15.9)		24	76	0.7	-
**No job exposure**	270	(84.1)		24	76	(0.3,1.5)	
**International travel (within 6 mos)**	102	(31.8)		34	66	2.0	2.0
**No travel**	219	(68.2)		20	80	(1.2,3.4)^**^	(1.1,3.6)*
**Sports participation (within 6 mos)**	119	(37.1)		35	65	2.1	2.5
**No sports**	202	(62.9)		18	82	(1.2,3.7)^**^	(1.4,4.4)^**^
**Child in daycare**	64	(19.9)		36	64	1.5	-
**No child in daycare**	257	(80.1)		21	79	(0.8,2.9)	
**Child <5 years old in home**	64	(20.0)		38	62	1.7	-
**No children <5 years old**	257	(80.0)		21	21	(0.9,3.2)	
**Child <18 years old in home**	159	(49.5)		31	69	1.6	-
**No children <18 years old**	162	(50.5)		17	83	(0.7,3.5)	
***Health-related exposures of household to skin infection***							
**Hospitalization (2 yrs)**	124	(38.6)	33		67	1.9	-
**No hospitalization**	197	(61.4)	19		81	(1.1,3.3)^**^	
**Surgery (within 6 mos)**	48	(15.0)	40		60	2.2	2.4
**No surgery**	273	(85.0)	22		78	(1.2,4.3)*	(1.2,4.8)*
**Antibiotic use (within 6 mos)**	165	(51.4)	34		66	3.0	2.7
**No antibiotic use**	156	(48.6)	14		86	(1.7,5.2)^***^	(1.5,4.8)^***^
***S. aureus*** ** positive**	138	(43.0)	30		70	1.4	-
***S. aureus*** ** negative**	183	(57.0)	20		80	(0.8,2.4)	
***Hygiene-related exposures of household to skin infection***							
**Shared towels**	60	(18.7)		38	62	2.0	1.9
**No shared towels**	261	(81.3)		21	79	(1.1,3.7)*	(1.0,3.7)*
**Shared razors**	36	(11.2)		36	64	1.7	
**No shared razors**	285	(88.8)		23	77	(0.8,3.5)	

* p≤.05, ^**^p<.01, ^***^p<.001.

### Microbiology of the *S. aureus* isolates

There was a low overall similarity score for the PFGE profiles of the 203 *S. aureus* positive specimens (similarity index 26%). One cluster of four methicillin susceptible isolates from three unrelated households fit the PFGE profile of USA800 (the pediatric clone).

Six (3%) of 203 *S. aureus* positive specimens were methicillin resistant; therefore, the community MRSA prevalence was 0.4%. Three MRSA positive specimens were SCC*mec* type III and three were SCC*mec* type IV; no specimens were USA300. The six individuals colonized with MRSA ranged in age from 29 to 76. Two had been hospitalized in the past two years, three were employed, one participant reported serious skin infection, and one reported antibiotic use.

### *S. aureus* transmission within the household

**Figure 1:**
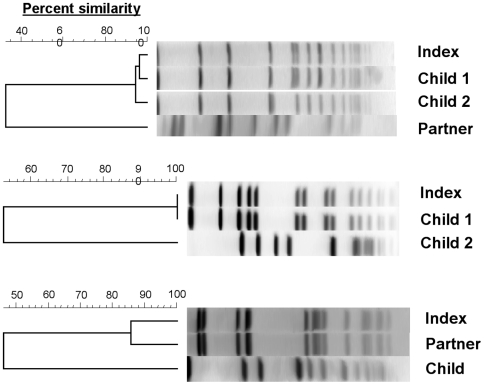
Dendrogram of isolates from three different households illustrating the presence of both similar and dissimilar isolates. Isolates >70% similar are considered closely related.

Among the 219 (68%) households containing more than one person, 46 (21%) had more than one household member colonized with *S. aureus*; two had four colonized household members, 10 had three, and 34 had two. Twenty-three households had similar strains (using a 70% cutoff of the Dice coefficient), and 30 had dissimilar strains of *S. aureus* (seven had both similar and dissimilar strains among *S. aureus* colonized individuals within their households, Figure 1). Trend analyses for intra-household transmission showed no increased risk for any of the exposures examined.

## Discussion

This population based survey is the first to integrate household level data and molecular epidemiology and finds significant and independent relationships linking community, health-related and hygiene exposures with serious skin infection at both the individual and the household level. This finding was not replicated in analyses that examined the relationship between these same exposures and *S. aureus* nasal colonization. However, the nasal colonization data did provide evidence of intra-household *S. aureus* transmission. The objective of this exploratory research was to determine if *S. aureus* colonization is a useful proxy measure to study disease transmission and infection in community settings; the results suggest that colonization is an incomplete proxy variable. In addition, the study also highlights the importance of the household unit as a significant community reservoir, since more than 40% of households were colonized and one quarter had household members with serious recent skin infections.

While increases have been observed in the community in both the prevalence and incidence of *S. aureus* infection, parallel increases in the incidence and prevalence of nasal colonization have not uniformly been observed [16], even among high risk populations [17]. Rather, *S. aureus* nasal colonization has been stable across both time and populations. Therefore, while *S. aureus* nasal colonization is an important marker of potential subsequent infection among hospital populations, it may not fully reflect *S. aureus* exposure and risk in the community, though it clearly plays a role. Ultimately however, *S. aureus* nasal colonization alone may not be a satisfactory proxy measure in the community setting [8].

Little is understood about the exact mechanisms of *S. aureus* transmission within the community, beyond a generic conceptual idea that transmission occurs through ‘contact.’ Research examining infectious disease transmission dynamics using social network methods has found that the prevalence of infection and the degree of contact infected individuals have with other group members, plays a significant role in both disease prevalence and incidence within a community [18]. This research began with the most ubiquitous and easily identified network in the community setting: the household, where more than 40% were found to be colonized. While it is not surprising that a higher percentage of households than individuals are colonized, the magnitude of colonization (>40%) suggests that households are an underappreciated and substantial community reservoir for infection.

As early as 1960, Roodyn [19] understood the importance of the household but observed “… that even in the comparative simplicity of the single household, the epidemiology of staphylococcal infections appears baffling.” This is the first study to address the household network on a large scale. These data found that more than two in every five households were colonized. Moreover, one fifth of multiperson households had multiple members colonized, half of whom were colonized with the same strain, suggesting at least some degree of intra-household transmission. Yet the data from this study suggest that the factors contributing to *S. aureus* infection are manifold and extend beyond the household.

The lack of strain similarity suggests that many strains are resident in the community and are regularly introduced into the household. These strains may then be transmitted either within the household or to extra-household contacts. Sexual transmission, both homosexual [20] and heterosexual [7], is a novel mechanism that is increasingly well documented and that may effectively spread pathogens. In addition, risk factors that introduce *S. aureus* into the household (*e.g.*, international travel, sports participation or recent surgery), are the same mechanisms through which individuals living in colonized households may re-introduce the pathogens to the community.

The role played by antibiotic use is deserving of special attention, since approximately one quarter of the more than 900 study participants had used antibiotics in the past six months, which translated to antibiotic usage by more than half of all randomly selected households. While antibiotic use independently conferred protection from *S. aureus* colonization among households, it was also independently associated with increased serious skin infections at both the individual and the household level. The data suggest that at least some usage may be unmonitored, given the high levels of self-reported non-prescription antibiotic use. Uncontrolled and perhaps inappropriate use of antibiotics has the potential to eventually increase resistance not only to methicillin, but to other first and second line antibiotics as well, allowing these increasingly antibiotic-resistant strains to circulate in the community. This phenomenon is already being observed [20].

Studies have documented considerable variation in the prevalence of methicillin-resistant *S. aureus* (MRSA) infections in different geographic regions [21], [22], [23]. While this study identified primarily susceptible *S. aureus* strains, there is no evidence to date to suggest that MRSA strains such as USA300, while perhaps more efficiently transmitted, are spread via different modalities. The presence or absence of the *mec* element does not appear to influence transmissibility [24]. The results from this randomly selected household sample suggest that the low background prevalence of MRSA is the sole limiting factor in large-scale colonization, since there is little to differentiate those colonized by *S. aureus* from those colonized by MRSA, or for that matter, those not colonized at all. Therefore, it is essential to identify mechanisms of transmission and other community reservoirs, in order to develop and implement interventions prior to a widespread dissemination of MRSA.

This study has limitations. The study population was largely Hispanic and therefore may not necessarily apply to other populations. Much of the data are self-reported and may underreport individual practices. There is evidence that underreporting occurred for socially undesirable factors (*e.g.*, illicit drug use). Although egocentric network data collection, whereby one person provides data for a group of individuals, is standard in social network research, individuals may underreport network member risk factors because of lack of knowledge. Underreporting would bias findings to the null. We cannot be certain that all skin infections were due to *S. aureus* or that the respondents recalled infections of the other household members although the bulk of skin infections are due to *S. aureus* [25]. In addition, RDD has been criticized for potential sample undercoverage due to the exclusion of cell-phone-only households. Only the nares were cultured; sampling of other body sites would likely have yielded a higher number of colonized participants [7], [8]. Alternatively prior antibiotic use may also have affected the rates of colonization in our sample. While some studies have documented limited nasopharyngeal penetration of many antibiotics, it remains possible that colonization was affected by the recent use of antibiotics [26]. Finally, despite the large number of individuals represented in this study, the household sample size was small. Larger community samples, as well as the inclusion of a more diverse sample, and innovative methodologies will be required to address current study limitations.

The multidisciplinary approach utilized in this study, combining epidemiology, social network methods and microbiology, facilitated an exploration of two separate lines of inquiry addressing *S. aureus* transmission within the community. The first line of inquiry identified significant relationships between several community, health-related and hygiene exposures and serious skin infection, but not *S. aureus* colonization. Results from the second line of inquiry suggest that households represent a significant *S. aureus* community reservoir, since more than 40% of households were colonized and 25% reported serious skin infection. The lack of association between *S. aureus* nasal colonization and serious skin infection underscores the need to explore alternative venues, environmental or body sites that may be crucial to transmission, as well as the importance of carrying out community based research independent of healthcare settings, in order to better understand *S. aureus* transmission dynamics in the community and to develop effective prevention strategies.
